# Dynamically stable and amplified circularly polarized excimer emission regulated by solvation of chiral co-assembly process

**DOI:** 10.1038/s41467-022-32714-1

**Published:** 2022-08-20

**Authors:** Yuxia Zhang, Hang Li, Zhongxing Geng, Wenhua Zheng, Yiwu Quan, Yixiang Cheng

**Affiliations:** 1 State Key Laboratory of Coordination Chemistry, Jiangsu Key Laboratory of Advanced Organic Materials, School of Chemistry and Chemical Engineering, Nanjing University, Nanjing, 210023 China; 2 Key Laboratory of High Performance Polymer Material and Technology of Ministry of Education, Department of Polymer Science and Engineering, School of Chemistry and Chemical Engineering, Nanjing University, Nanjing, 210023 China

**Keywords:** Molecular self-assembly, Self-assembly, Organic molecules in materials science

## Abstract

Chiral supramolecular assembly has been assigned to be one of the most favorable strategies for the development of excellent circularly polarized luminescent (CPL)-active materials. Herein, we report our study of an achiral boron-containing pyrene (Py)-based chromophore (PyBO) as a circularly polarized excimer emission (CPEE) dye induced by chiral co-assemblies containing chiral binaphthyl-based enantiomers (*R*/*S*-M). Chiral co-assembly *R*/*S*-M-(PyBO)_4_ fresh film spin-coated from toluene solution can exhibit orderly nanofibers and strong green CPEE (λ_em_ = 512 nm, *g*_em_ = ±0.45, *Φ*_FL_ = 51.2 %) resulting from an achiral PyBO excimer. In contrast, only a very weak blue CPL was observed (λ_em_ = 461 nm, *g*_em_ = ±  0.0125, *Φ*_FL_ = 19.0 %) after 187 h due to PyBO monomer emission as spherulite growth. Interestingly, this kind of chiral co-assembly *R*-M-(PyBO)_4_-T film from tetrahydrofuran (THF) solution retains uniform morphology and affords the most stable and strongest CPEE performance (λ_em_ = 512 nm, *g*_em_ = + 0.62, *Φ*_FL_ = 53.3 %) after 10 days.

## Abstract (web summary)

Chiral supramolecular assembly is an important strategy for the development of excellent circularly polarized luminescent (CPL)-active materials, but often they suffer from low quantum yield and luminescence dissymmetry factor. Herein, the authors we report a chiral coassembly process of achiral pyrene-based dyes leading to chiral excimers emitting with a high dissymmetry factor.

## Introduction

In the past several years, much work has focused on circularly polarized luminescence (CPL) materials because of their potential applications in 3D optical displays, information encryption, and chiral sensing^1–5^. These sophisticated CPL-active materials inevitably involve two common problems—their low quantum yield (*Ф*_FL_) and the luminescence dissymmetry factor (*g*_em_). With the rapid development of chiral supramolecular chemistry, some simple chiral organic molecules can self-assemble or co-assemble as highly regular arrangements and can generate an orderly helical superstructure of chiral building blocks during the host–guest combination process. This is greatly beneficial to the amplification of CPL signals through intramolecular or intermolecular chirality transfer mechanisms^6–10^. As notably, excimers have been applied widely in the fields of organic optoelectronics, organic lasers, chemical and biological sensing, cell imaging, and stimulus response due to their large redshift and the absence of fine structure^11–15^. Increasing attention has been paid to chiral supramolecular excimers as circularly polarized excimer emission (CPEE) materials, whose behavior is attributed to asymmetrically steric architectures in their excited states^16–23^. In 2016, our group^17^ observed chiral 1,2-diaminocyclohexane-based molecules incorporating 1,8-naphthalimide fluorophores could emit strong blue CPEE signals (*g*_lum_ = ±0.037, *λ*_em_ = 470 nm) from the 1,8-naphthalimide excimer in MeOH solution. Reversed CPL signals were detected however in the aggregated state (THF/H_2_O = 5/95) due to the regular and orderly alignment of the aggregates. Recently, Zhu and Liu^20^ achieved excellent CPL performance using three symmetric (P1–P3) and one asymmetric (B) chiral V-shaped pyrenes through molecular conformation-guided chiral hexagonal supramolecular packing and various helical nanoarchitectures. The amplified CPEE signals they obtained could be further promoted by the efficient chirality transfer of intra- or intermolecular chiral excimers.

It is known that pyrene (Py) derivatives, one of the most excellent supramolecular self-assembly or co-assembly luminophores, are regarded as the suitable and promising candidates for excimer emission due to their rigid, planar and conjugated structures^24,25^. Recently, chiral Py-based self-assemblies and co-assemblies have been selected as CPEE-active dyes through the regulation of intra- or intermolecular π-π aromatic stacking^20,26–28^. In 2022, Shigemitsu et al.^28^ developed a series of Py-cyclodextrins (PCDs) grafted multiple Py units on a cyclodextrin scaffold to produce spatially restricted excimers whose properties originate from their steric hindrance and cumulative interactions, and bright green CPEE signals (*λ*_em_ = 478–486 nm, |*g*_lum_|≈10^−2^) was observed in CH_2_Cl_2_ (DCM) solution. This work also found that the stacking manner of Py-based excimers could give rise to a reversed CPEE by changing the number of carbon atoms in the linker of PCDs, an odd-even effect. To date, most of these chiral excimers have been in solution^28–30^, gel^31^, or the crystalline^32^ or powder state^33^, and no report has focused on the stability and the mechanism of formation of chiral excimers. Consequently, it is of great significance to make in-depth insights into the dynamic stability of chiral supramolecular excimers in the spin-coated film states.

**Fig. 1:**
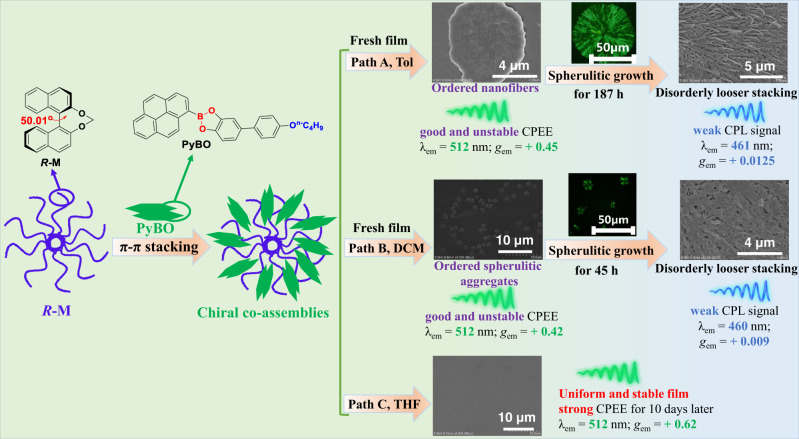
Schematic representation. Schematic representation of dynamic CPEE based on the chiral co-assembly process.

Axial chiral binaphthyl-based derivatives can trigger the amplification effect of intrinsic molecular chirality and chiral induction ability upon the anchored dihedral angle due to the highly rigid conformations^34–39^. In 2020, Takaishi’s group^27^ designed a chain of Py-based excimers sandwiched by axially chiral 1,1′-binaphthyls via ester linkers and observed the reversed CPEE signals in nonpolar and polar solvents, respectively. The exchange of (−)-CPEE and (+)-CPEE was closely related to the inversion of excimer chirality caused by the presence or absence of intermolecular hydrogen bonds in the excited state. Our group recently carried out a series of experiments on the amplified CPL emission promoted by the intermolecular chiral transfer of chiral binaphthyl inducers with the anchored dihedral angle^9,10,40–42^. In this paper, we found a dynamically stable and amplified CPEE of an achiral Py-based dye regulated through the solvation effect of chiral co-assembly process. Chiral co-assemblies were rationally constructed by choosing an achiral boron-containing Py-based chromophore (PyBO) as the CPEE dye and chiral binaphthyl enantiomers (*R*/*S*-M) with an anchored dihedral angle as chiral inducers (Fig. 1). Chiral co-assemblies *R*-M-(PyBO)_4_ and *R*-M-(PyBO)_4_-D films were spin-coasted from toluene solution (Path A) and DCM solution (Path B), respectively. Interestingly, these two *R*-M-(PyBO)_4_ and *R*-M-(PyBO)_4_-D fresh films exhibited good CPEE behaviors (*λ*_em_ = 512 nm, *g*_em_ = + 0.45 or +0.42) in the nanofibers or spherulitic aggregates during the process of chiral supramolecular assembly. This kind of CPL signal however blue-shifted gradually from 512 to 461 nm and accompanied by a decrease of *g*_em_ to +0.0125 and +0.009 at 187 h and 45 h resulting from spherulitic growth. As is evident from Path C in Fig. 1, we strikingly observed that chiral co-assembly *R*-M-(PyBO)_4_-T film spin-coated from THF solution not only retained a uniform morphology, but also remained the most stable and strongest CPEE (*λ*_em_ = 512 nm, *g*_em_ = +0.62, *Φ*_FL_ = 53.3%) for 10 days later. This is the largest *g*_em_ value of CPEE-active materials recorded to date (Supplementary Table 2). This work revealed that such dynamically stable CPEE behavior is directly related to the solvation effect during the chiral co-assembly process.

## Results and discussion

### Photophysical properties of *R*-M, PyBO and chiral co-assemblies *R-*M-PyBO

**Fig. 2:**
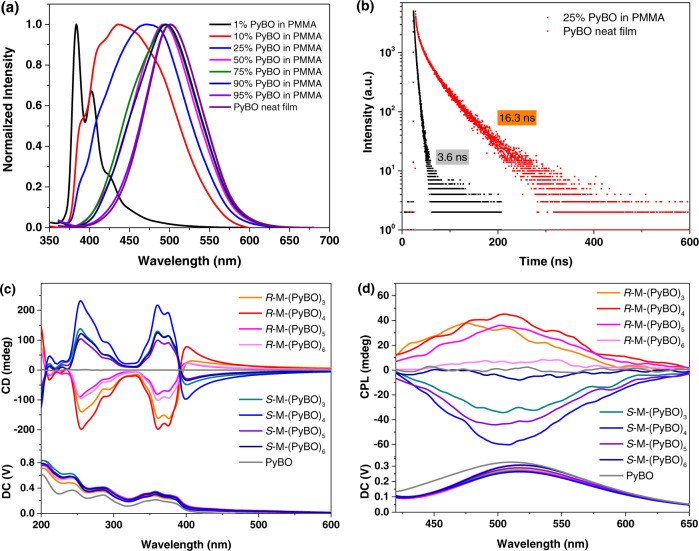
Optical properties and chiral optical properties. **a** FL spectra of the blend films doping PyBO in matrix material PMMA (wt % of PyBO from 1 to 95%) and PyBO neat film (*λ*_ex_ = 330 nm); **b** The fluorescence decay of the blend film doping 25 wt% PyBO in PMMA (*λ*_em_ = 460 nm) and PyBO neat film (*λ*_em_ = 500 nm); **c** CD spectra and **d** CPL spectra of chiral co-assemblies *R/S*-M-PyBO at different molar ratios (*λ*_ex_ = 330 nm). All spin-coated films from toluene solutions (10 mg/mL) on quartz plates (1 cm × 3 cm) (1000 N/s, 30 s).

Chiral co-assembly *R-*M-PyBO films were spin-coated from toluene solutions at different molar ratios according to the methods described in the Methods part. The UV–vis absorption and FL emission spectra of achiral PyBO and *R-*M-PyBO were measured in their spin-coated films. PyBO showed two absorption peaks at 237 and 270 nm from the π–π* transition of the Py moiety and one peak at 357 nm from the conjugated structure. And the emission peak is located at 501 nm (Fig. 2a). As shown in Fig. 2a, the blend film doped with 1 wt% of PyBO in PMMA had two FL peaks at 383 and 402 nm, which can be assigned to the typical emission of the Py monomer^43^. We clearly observed the FL emission wavelength of PyBO appeared with a continuous red-shift to 501 nm as the doping content increased from 1 wt% to 95 wt%, indicating the formation of Py excimer. In terms of their fluorescence decays (Fig. 2b), the Py-based excimer has a longer luminescent lifetime and higher quantum yield (*τ* = 16.3 ns, *Φ*_F_ = 49.7%) than its corresponding monomer (*τ* = 3.6 ns, *Φ*_F_ = 20.1%). In contrast, *R-*M-PyBO always had the same absorption and excimer emission (*λ*_em_ = 500 nm) as PyBO excimer (Supplementary Fig. 2).

### CD and CPL properties of chiral co-assemblies *R/S*-M-PyBO

We investigated the CD and CPL properties of chiral co-assemblies *R/S*-M-PyBO in the spin-coated films from toluene solutions. (*R/S*-M)_2_-PyBO at a 2:1 molar ratio of *R/S*-M with PyBO has three Cotton peaks at 207, 235 and 330 nm similar to the chiral inducer *R/S*-M, but a weak mirror-image Cotton peak was detected at 361 nm, which could be regarded as the absorption of the achiral PyBO moiety (Supplementary Fig. 3a). As increasing the molar ratio of PyBO, the original peaks gradually weakened and finally completely disappeared. But the new mirror-image peaks situated at 254, 361, 374, and 401 nm became stronger and stronger indicating the formation of chiral co-assembly through intermolecular π–π stacking interactions (Fig. 2c and Supplementary Fig. 3a). Obviously, *R/S*-M-(PyBO)_4_ at a 1:4 molar ratio had the strongest CD signal at 254 nm (|*g*_ab_| = 1.47 × 10^−2^), 361 nm (|*g*_ab_| = 2.15 × 10^−2^), 375 nm (|*g*_abs_ | = 2.25 × 10^−2^) and 401 nm (|*g*_ab_| = 3.05 × 10^−2^), which coincides well with the absorption of PyBO and confirms the effective chiral transfer from chiral inducer *R/S*-M to the achiral PyBO. Most interestingly, this *R/S*-M-(PyBO)_4_ can emit bright green CPEE signals (*λ*_em_ = 512 nm, |*g*_e_| = 1.49 × 10^−2^) stemming from achiral PyBO (Fig. 2d, Supplementary Figs. 3b and 4).

### CPL properties of *R/S*-M-(PyBO)_4_ with different film thicknesses

**Fig. 3:**
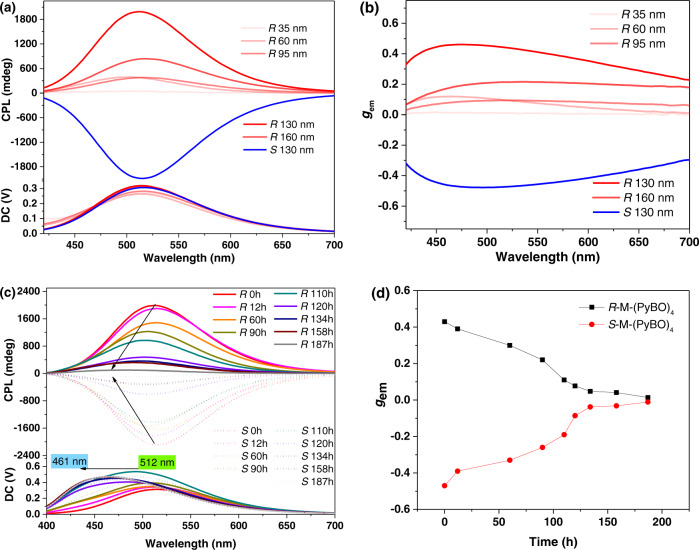
CPL properties at different conditions. **a** CPL spectra and **b** the *g*_em_ values of chiral co-assembly *R*/*S*-M-(PyBO)_4_ prepared by spin-coating in toluene solutions at different concentrations; **c** CPL spectra and **d** the *g*_em_ values of *R*/*S*-M-(PyBO)_4_ vs time (h) in air (the spectra were obtained by superimposing the front and back tests, *λ*_ex_ = 330 nm).

And then we also explored the influence of the spin-coated film thickness on the CPEE of *R/S*-M-(PyBO)_4_. As is seen in Fig. 3a, b and Supplementary Table 1, R*/S*-M-(PyBO)_4_ had the strongest CPEE signal (*λ*_em_ = 512 nm, |*g*_em_| = 0.45, *Φ*_F_ = 51.2%) at 130 nm thickness. The thickness dependence of the *g*_em_ values indicated that the intrinsic CPL can be affected by extrinsic factors, such as the differential absorption of CP light (i.e., circular dichroism) or the birefringent properties of chiral materials^44,45^. This result further certified that the initial chirality could be strongly amplified by the anisotropy of system. Moreover, almost no change was detected in the CPL spectra of *R/S*-M-(PyBO)_4_ after rotation or flipping (on the front or back sides), showing that there is less linear dichroism or linear birefringence effect in every measured process (Supplementary Fig. 5)^46^. However, it can be clearly observed that the CPEE of *R/S*-M-(PyBO)_4_ gradually blue-shifted by as much as 51 nm to a fixed monomer emission (Fig. 3c and Supplementary Fig. 6–8) and accompanied by the reduced |*g*_em_| values from 0.45 to 0.0125 after 187 h, which can be attributed to the unstable excimer of *R/S*-M-(PyBO)_4_ from toluene solution in air (Fig. 3c, d).

### POM properties of *R/S*-M, PyBO and *R*-M-(PyBO)_4_

**Fig. 4:**
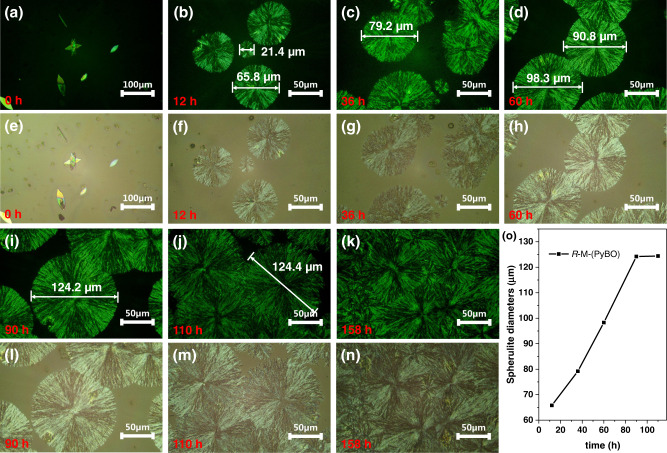
POM and optical microscope images. **a**–**d**, **i**–**k** POM images of *R*-M-(PyBO)_4_; **e**–**h**, **l**–**n** Optical microscope images of *R*-M-(PyBO)_4_; **o** Plot of spherulite diameters of *R*-M-(PyBO)_4_ *vs* time (h) in air. All spin-coated films from toluene solution (40 mg/mL) on quartz plates (1 cm × 3 cm) (1000 N/s, 30 s).

In order to fully understand the CPEE stability, we further tested the spin-coated film morphologies of the chiral inducer *R/S*-M, the achiral PyBO and the chiral co-assembly *R*-M-(PyBO)_4_ in toluene solutions by using polarized optical microscope (POM) and optical microscope. In terms of POM images (Supplementary Fig. 9), chiral *R/S*-M showed two flower-like oriented crystallinities (clockwise rotation for *R*-type and counterclockwise rotation for *S*-type after 20 min), but irregular leaf crystallinity for achiral PyBO after 12 h (Supplementary Fig. 10). Interestingly, *R*-M-(PyBO)_4_ subsequently formed a specially spherulitic morphology with a typical black cross extinction texture after 12 h (Fig. 4a, b). And this spherulite diameter increased from 21.4 to 124.2 µm as the crystallization time extended to 90 h, which is due to the fact that fine crystals grew in a certain space and then collided with each other (Fig. 4). In addition, we also found that the CPL wavelength of *R*-M-(PyBO)_4_ blue-shifted from the unstable excimer emission (512 nm) to the fixed monomer emission (461 nm) until the crystallization finally stopped growth after 187 h (Supplementary Figs. 7 and 11), which led to the decreased CPL signal. We proposed that crystallization process could control the critical nucleation size of the crystallites until spherulite growth tends to reach an equilibrium at the energetically favorable state and then causes the disappearance of the excimer^47^.

### CPL properties of *R*-M-(PyBO)_4_-D and *R*-M-(PyBO)_4_-T

**Fig. 5:**
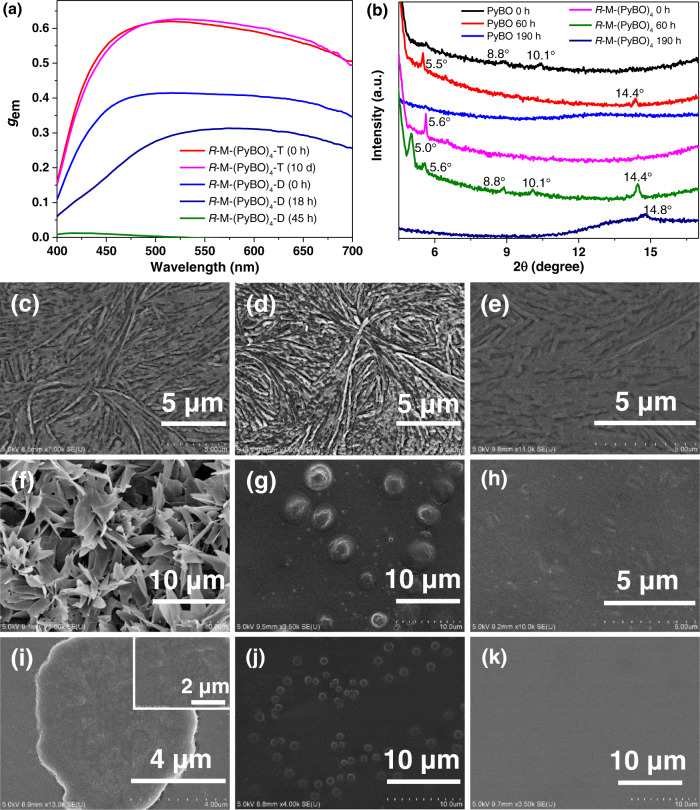
Chiral optical properties, the morphology study, and XRD patterns. **a** The *g*_em_ values of chiral co-assemblies *R*-M-(PyBO)_4_-D/T (*λ*_ex_ = 330 nm); **b** XRD patterns of PyBO and *R*-M-(PyBO)_4_ films spin-coated from toluene solution; the SEM images of **c**–**e** *R*-M fresh films and **f**–**h** PyBO fresh films spin-coated from toluene, DCM, and THF solutions, respectively; **i**–**k** the SEM images of *R*-M-(PyBO)_4_ and *R*-M-(PyBO)_4_-D/T fresh films. All spin-coated films from 40 mg/mL solutions on quartz plates (1 cm × 3 cm) (1000 N/s, 30 s).

It is known that solvation effect plays a pivotal role in the formation of crystal nucleus and spherulite growth. In this research, two kinds of chiral co-assembly of *R*-M-(PyBO)_4_ films were spin-coated using DCM and THF solutions both at a concentration of 40 mg/mL (defined as *R*-M-(PyBO)_4_-D and *R*-M-(PyBO)_4_-T), respectively. As shown in Fig. 5a, Supplementary Figs. 12a and 14, *R*-M-(PyBO)_4_-D fresh film had a good CPEE signal (*λ*_em_ = 512 nm, *g*_em_ = +0.42) in accordance with *R*-M-(PyBO)_4_ from toluene solution at smaller spherulite size (10 µm), indicating that small spherulites have no influence on CPEE. As the sequent growth of spherulite size, the CPEE of *R*-M-(PyBO)_4_-D completely disappeared and transferred as a very weak CPL emission from the monomer PyBO (*λ*_em_ = 460 nm, *g*_em_ = + 0.009) after 45 h. On the contrary, the crystallization process of *R*-M-(PyBO)_4_-T was not observed until 10 days later (Supplementary Fig. 15), which can be attributed to the good solubility for both *R*-M and PyBO in THF solution. Especially, *R*-M-(PyBO)_4_-T can emit the strongest CPEE signal (*λ*_em_ = 512 nm, *g*_em_ = +0.62) according to the published reports (Fig. 5a and Supplementary Table 2), and this CPEE signal could be still detected after 10 days without linear dichroism or linear birefringence effect in every measured process (Supplementary Fig. 12b). It was assumed that the dynamical stability and amplified effect of CPEE can be successfully facilitated by solvation of chiral co-assembly process due to the different solubilities on the chiral inducer and excimer. In addition, *R*-M-(PyBO)_4_-T also showed the thickness dependence of *g*_em_ values and the highest *g*_em_ value (+0.62) at 270 nm (Supplementary Fig. 13).

### The morphology study and XRD patterns of *R*-M, PyBO, and *R*-M-(PyBO)_4_

To deeply study the chiral transfer mechanism from chiral inducer to achiral excimer, the scanning electron microscope (SEM) micromorphologies of *R*-M-(PyBO)_4_, *R*-M-(PyBO)_4_-D, and *R*-M-(PyBO)_4_-T films were spin-coated by using toluene, DCM, and THF solutions, respectively. All SEM morphologies of *R*-M films showed the same regular nanofibers (Fig. 5c–e). The surfaces of PyBO fresh films became smoother and smoother, and the aggregate images gradually changed from sheets to flat accumulations as the solvent polarity increased (Fig. 5f–h). As expected, it can be obviously observed that the morphology of *R*-M-(PyBO)_4_ fresh film showed many nanofibers and then entangled each other as the circular aggregates to act as a crystal nucleus for the spherulite growth (Fig. 5i). In contrast, *R*-M-(PyBO)_4_-D fresh film is prone to spherical aggregates (Fig. 5j). However, *R*-M-(PyBO)_4_ and *R*-M-(PyBO)_4_-D gradually generated the nanocrystallites, and then looser stacking appeared at the critical nucleation size after 187 h and 45 h respectively, which finally led to the disappearance of the chiral excimer (Supplementary Figs. 16h and 17h). Most importantly, the smooth and uniform morphology for *R*-M-(PyBO)_4_-T remained for 10 days later (Fig. 5k and Supplementary Fig. 18). Therefore, it appeared that the amplified CPEE behavior can be adjusted by the dynamically stable chiral co-assembly during the solvation of supramolecular assembly.

As is evident from the X-ray diffraction (XRD) patterns of the spin-coated thin-films from toluene solution, only very weak diffraction peaks of PyBO were observed for the fresh film. Interestingly, two main diffraction peaks were centered at 2*θ* = 5.5° and 14.4° at 60 h (see below Fig. 5b), but completely disappeared at 190 h, which coincides well with the POM and SEM morphologies. *R*-M-(PyBO)_4_ film had a new diffraction peak at 5.0° at 60 h indicating the formation of a chiral co-assembly. But only the diffraction peak (2*θ* = 14.8°) still remained at 190 h, which may be due to the change of the aggregate stacking type as spherulite growth. Most importantly, *R*-M-(PyBO)_4_-T film from THF solution always retained the stable broad diffraction peaks at 12.3° for 10 days later, further demonstrating the dynamic stability of uniform morphology without the spherulite growth or crystallization (Supplementary Fig. 19). It is also the intrinsic reason for the stable CPEE of *R*-M-(PyBO)_4_-T film regulated by THF solvation effect.

In summary, two chiral co-assemblies *R*-M-(PyBO)_4_ and *R*-M-(PyBO)_4_-D films were spin-coated from toluene and DCM, respectively. And their unstable CPEE signals gradually disappeared at 187 h and 45 h respectively as spherulitic growth proceeded. In contrast, the chiral co-assembly *R*-M-(PyBO)_4_-T still exhibited a uniform morphology and emitted the dynamically most stable and strongest CPEE signal (*λ*_em_ = 512 nm, *g*_em_ = +0.62) 10 days later. This is attributed to their good solubility on both the chiral inducer (*R*-M) and the achiral excimer (PyBO). Therefore, this work has provided a deep insight into the dynamic stability of chiral supramolecular excimers regulated by solvation effect in film states.

## Methods

### The prepared methods of spin-coated films

10 mg of PyBO and *R*/*S*-M were dissolved in 1 mL toluene. Then, the *R*/*S*-M solution was dropped into the above PyBO solutions at various doping ratios. The co-assemblies were collected from the above-mixed solutions by spin coating (1000 N/s, 30 s) on quartz plates (1 cm × 3 cm) after toluene was naturally evaporated at room temperature.

## Supplementary information

Supplementary Information

## Data Availability

The authors declare that all other data supporting the findings of this study are available within the article and Supplementary Information files, and also are available from the corresponding author upon request. Source data are provided in this paper.

## Source data

Source Data
