# Supplementary material for: Dynamically stable and amplified circularly polarized excimer emission regulated by solvation of chiral co-assembly process
Source: Nat Commun. 2022 Aug 20;13:4905. doi: 10.1038/s41467-022-32714-1 (PMC9392786; doi:10.1038/s41467-022-32714-1)
Supplement: Supplementary file 1 — Supplementary Information [file 41467_2022_32714_MOESM1_ESM.pdf]

Supplementary Information

Dynamically Stable and Amplified Circularly Polarized Excimer Emission Regulated by Solvation of Chiral Co-assembly Process

*Yuxia Zhang,[1] Hang Li,[1] Zhongxing Geng,[1,2] Wen-hua Zheng, *[1] Yiwu Quan*[2] and Yixiang Cheng*[1]*

1. State Key Laboratory of Coordination Chemistry, Jiangsu Key Laboratory of Advanced Organic Materials, School of Chemistry and Chemical Engineering, Nanjing University, Nanjing 210023, China. E-mail: yxcheng@nju.edu.cn. E-mail: wzheng@nju.edu.cn.

2. Key Laboratory of High Performance Polymer Material and Technology of Ministry of Education, Department of Polymer Science and Engineering, School of Chemistry and Chemical Engineering, Nanjing University, Nanjing 210023, China. E-mail: quanyiwu@nju.edu.cn

Table of Contents

1. Measurements and materials.
2. Synthesis and characterizations.
3. UV-*vis* and FL spectra of *R*-M-PyBO.
4. CD and CPL spectra of *R/S*-M-PyBO.
5. The g_{em} values of different film thickness of *R/S*-M-$(PyBO)_4$.
6. CPL spectra of *R/S*-M-$(PyBO)_4$.
7. The pictures under UV light and plot of maximum emission wavelength of *R/S*-M-$(PyBO)_4$ versus time.
8. CPL spectra of *R/S*-M-$(PyBO)_4$ at different time.
9. POM and optical microscope images of *R/S*-M and PyBO films spin-coated from toluene solutions.
10. The plot of maximum emission wavelength of *R*-M-$(PyBO)_4$ versus spherulite diameters.
11. CPL spectra of *R*-M-$(PyBO)_4$-D/T.
12. POM and optical microscope images of *R*-M-$(PyBO)_4$-D/T.
13. SEM images.
14. XRD patterns.
15. Comparison of the part of chiral excimer system.
16. ^1H and ^{13}C NMR Spectra of Compounds.
17. Reference.

1. Measurements and materials.

All NMR spectra were obtained by using a Bruker (400 MHz) spectrometer. Chemical shifts were recorded as parts per million (ppm, δ) relative to tetramethylsilane (δ 0.00) or chloroform (δ = 7.26, singlet). ^{1}H NMR splitting patterns are designated as singlet (s), doublet (d), triplet (t), multiplets (m) and etc. Ultraviolet-visible (UV-*vis*) spectra were measured by using a Shimadzu UV-3600 spectrophotometer. Fluorescence spectra were tested at 1200 nm/min of scan speed from a HORIBA Scientific FluoroMax-4 Spectrofluorometer. X-ray diffraction (XRD) was measured by using Rigaku D/Max-2500 X-ray diffractometer (Japan) with Cu/Kα radiation (λ = 1.5406 Å), which was operated at a voltage of 40 kV and a current of 200 mA. The absolute PL quantum yield were measured by a HORIBA Fluorolog-3 3D-Spectrofluorometer. Circular dichroism (CD) spectra were performed on a JASCO J-810 spectropolarimeter with 200 nm/min of scan speed. CPL spectra were collected on a JASCO CPL-300 spectrofluoropolarimeter. In the CPL measurements, scan speed was 200 nm/min, number of scans was 1, and slit width was 3000 μm. Scanning electron microscope (SEM) images were taken in Hitachi S-4800 field emission scanning electron microscopy. The liquid crystalline textures were investigated and photographed using liquid crystal cells with a polarized optical microscope (POM) equipped with a Leitz-350 heating stage and an associated Nikon (D3100) digital camera. All solvents and reagents were commercially available A.R. grade.

2. Synthesis and characterizations

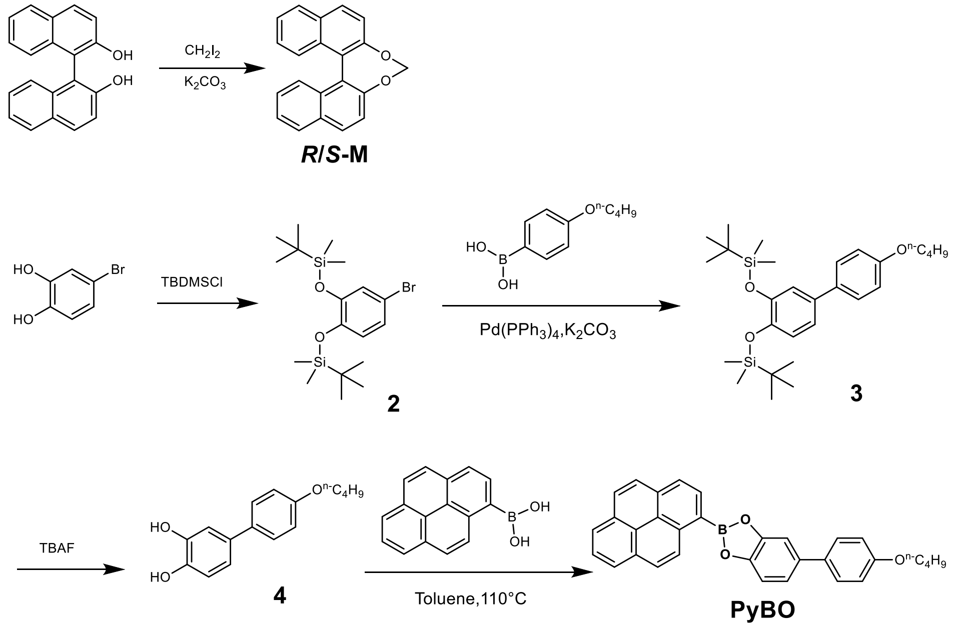

Supplementary Fig. 1. Synthetic routes. The synthetic routes of *R*/*S*-M and PyBO.

a) Synthesis and characterizations of *R-/S*-M.

R/*S*-M were synthesized according to the literature. [1,2] To a solution of the *R*/*S*-BINOL (1.0 equiv), K_2CO_3 (6.0 equiv) and CH_3CN was added CH_2I_2 (3.0 equiv) at room temperature. The mixture solution was refluxed at 80 °C for overnight. After the reaction completed (detected by TLC) and cooled to the room temperature, it was filtered to remove excess K_2CO_3, and the solvent removed under reduced pressure. The crude product was purified by flash column chromatography (silica gel) to afford the compound *R*/*S*-M (95% yield). ^{1}H NMR (400 MHz, $CDCl_3$): δ 8.01-7.94 (m, 4H), 7.55-7.44 (m, 6H), 7.34-7.30 (m, 2H), 5.72 (s, 2H); ^{13}C NMR (100 MHz, $CDCl_3$): δ 151.3, 132.2, 131.8, 130.4, 128.4, 126.9, 126.1, 126.0, 125.0, 121.0, 103.2; HRMS (ESI): m/z Calculated for $C_{21}H_{14}O_2Na^+$ [M+Na$^+$]: 321.0891; Found 321.0896.

b) Synthesis and characterizations of 2. [3]

To a solution of the 4-bromocatechol (1.0 equiv) in CH_2Cl_2 was slowly added imidazole (2.4 equiv) at 0°C. After stirring for 20 min, to the mixture was slowly added tert-Butyldimethylsilyl chloride (3.0 equiv) at the same temperature. After being stirred for 4h at room temperature (detected by TLC), the reaction mixture was poured into water to quench the reaction and extracted with CH_2Cl_2. The combined organic layer was washed with brine, dried over Na_2SO_4 and the solvent removed under reduced pressure. The crude product was purified by flash column chromatography (silica gel) to afford the compound **2** as colorless oil (91% yield). ^{1}H NMR (400 MHz, $CDCl_3$): δ 6.95 (d, *J* = 2.4 Hz, 1H), 6.93-6.90 (m, 1H), 6.69 (d, *J* = 8.4 Hz, 1H), 0.98 (s, 9H), 0.97 (s, 9H), 0.20 (s, 6H), 0.19 (s, 6H). ^{13}C NMR (100 MHz, $CDCl_3$): 148.1, 146.6, 124.5, 124.4, 122.4, 112.9, 26.2, 26.1, 18.7, 18.6, -3.9, -4.0; HRMS (ESI): m/z Calculated for $C_{18}H_{34}BrO_2Si_2^+$ [M+H$^+$]: 417.1276; Found 417.1271.

c) Synthesis and characterizations of 3 and 4.

Under Ar atmosphere, to a solution of the compound **2** (1.0 equiv), corresponding boronic acid/esters (1.5 equiv) and $Pd(PPh_3)_4$ (4 mol%) were added toluene (7 mL/mmol), aqueous Na_2CO_3 solution (2.0 M, 3.2 mL/mmol) and ethanolic (3.2 mL/mmol), and the resulting suspension was refluxed until the reaction was completed (detected by TLC). After cooling to room temperature, EA and H_2O were added. The organic phase was separated, washed with brine, dried over Na_2SO_4 and the solvent removed under reduced pressure. The crude product was purified by flash column chromatography (silica gel) to afford the compound **3** (85% yield). The compound **3** was directly diluted in anhydrous THF. TBAF was added (1.0 M in THF, 3.0 equiv) the mixture at ice-water and the reaction mixture was stirred for 4 h at room temperature (detected by TLC). The reaction

was quenched by addition of MeOH, and the solvent was removed under reduced pressure. The crude product was recrystallized by using DCM/PE afford the compound **4** (>90% yield). ^{1}H NMR (400 MHz, $CDCl_3$): *δ* 7.42 (d, *J* = 8.8 Hz, 2H), 7.07 (d, *J* = 2.4 Hz, 1H), 7.00 (dd, *J* = 8.0, 2.0 Hz, 1H), 6.94-6.89 (m, 3H), 5.17 (s, 1H), 5.09 (s, 1H), 3.99 (t, *J* = 6.4 Hz, 2H), 1.82-1.75 (m, 2H), 1.55-1.46 (m, 2H), 0.99 (t, *J* = 7.2 Hz, 3H); ^{13}C NMR (100 MHz, $CDCl_3$): *δ* 158.4, 143.7, 142.6, 134.5, 133.1, 127.7, 119.4, 115.7, 115.6, 114.8, 113.9, 67.9, 31.4, 19.3, 13.9; HRMS (ESI): m/z Calculated for $C_{16}H_{19}O_3^+$ [M+H$^+$]: 259.1329; Found 259.1337.

d) Synthesis and characterizations of PyBO.

1-Pyrenylboronic acid (1.0 equiv, 100 mg) and compound **4** were dissolved in dry toluene (5 mL) and the mixture was refluxed at 110 °C in dark conditions for 48 h (the fluorescence of the reaction mixture became green-yellow color from blue-purple color). The toluene was removed under reduced pressure and the crude product was washed by using n-hexane to afford PyBO as solid. ^{1}H NMR (400 MHz, $CDCl_3$): *δ* 9.24 (d, *J* = 9.2 Hz, 1H), 8.84 (d, *J* = 7.6 Hz, 1H), 8.29-8.23 (m, 4H), 8.15 (dd, *J* = 26.8, 8.8 Hz, 2H), 8.06 (t, *J* = 7.6 Hz, 1H), 7.62 (d, *J* = 1.6 Hz, 1H), 7.57-7.54 (m, 2H), 7.46 (d, *J* = 8.4 Hz, 1H), 7.37 (dd, *J* = 8.4, 2.0 Hz, 1H), 7.02-6.99 (m, 2H), 4.03 (t, *J* = 6.8 Hz, 2H), 1.85-1.78 (m, 2H), 1.58-1.49 (m, 2H), 1.01 (t, *J* = 7.2 Hz, 3H); ^{13}C NMR (100 MHz, $CDCl_3$): *δ* 158.7, 149.0, 147.6, 136.7, 136.6, 134.4, 134.3, 133.3, 131.1, 130.7, 129.3, 128.7, 128.2, 127.4, 127.3, 126.0, 125.9, 125.8, 124.5, 124.4, 124.3, 121.5, 114.9, 112.5, 111.1, 67.8, 31.4, 19.3, 13.9; HRMS (ESI): m/z Calculated for $C_{32}H_{26}BO_3^+$ [M+H$^+$]: 469.1970; Found 469.1976.

3. UV-*vis* and FL spectra of *R*-M, PyBO and *R*-M-PyBO.

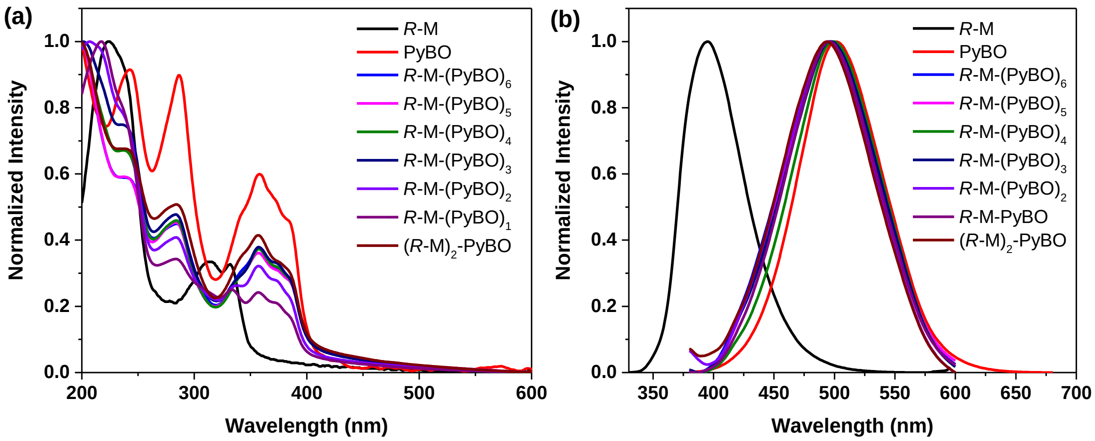

Supplementary Fig. 2. Optical properties. (a) UV-*vis* spectra and **(b)** FL spectra of *R*-M, PyBO and *R*-M-PyBO in the spin-coated film (λ_{ex} = 300 nm for *R*-M, λ_{ex} = 360 nm for PyBO and *R*-M-PyBO).

4. CD and CPL spectra of *R/S*-M-PyBO.

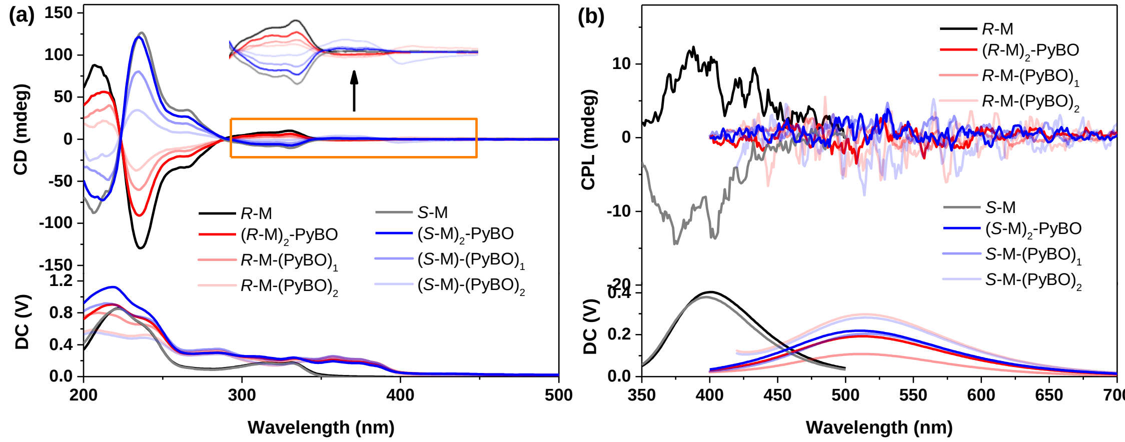

Supplementary Fig. 3. Chiral optical properties. (a) CD spectra and **(b)** CPL spectra of *R/S*-M-PyBO with different molar ratios in the spin-coated film (λ_{ex} = 280 nm for *R/S*-M, λ_{ex} = 330 nm for *R/S*-M-PyBO).

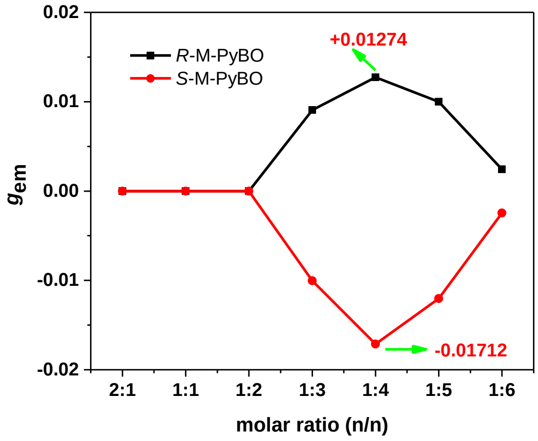

Supplementary Fig. 4. Blend g_{em} values. Plot of blend g_{em} values *vs* the doped concentration molar ratio of *R/S*-M-PyBO in the spin-coated film.

5. The g_{em} values of different film thickness of *R/S*-M-(PyBO)$_4$.

Supplementary Table 1 the g_{em} values of different film thickness of *R/S*-M-(PyBO)$_4$.

Concentration (mg/mL)[a]	Film Thickness (nm)	g_{em}
10 (*R*)	35	+ 0.015
20 (*R*)	60	+ 0.110
30 (*R*)	95	+ 0.092
40 (*R*)	130	+ 0.446
50 (*R*)	160	+ 0.210
40 (*S*)	130	- 0.475

[a] The concentration of the toluene solution of *R/S*-M-(PyBO)$_4$

6. CPL spectra of ***R/S*-M-(PyBO)$_4$.**

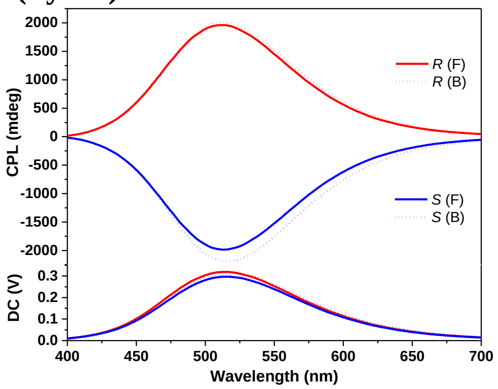

Supplementary Fig. 5. CPL spectra of *R/S*-M-(PyBO)$_4$. CPL spectra of *R/S*-M-(PyBO)$_4$ fresh film at 130 nm thickness (the films facing toward the detector: Frontward, F; away from the detector: Backward, B) (λ_{ex} = 330 nm).

7. The pictures under UV light and plot of maximum emission wavelength of *R/S*-M-(PyBO)$_4$ versus time.

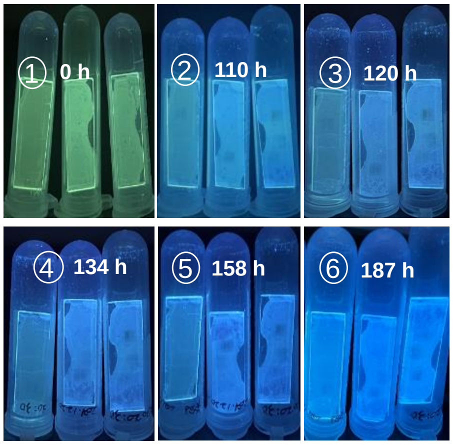

Supplementary Fig. 6. The pictures under UV light. The pictures of PyBO (left), *R*-M-(PyBO)$_4$ (middle) and *S*-M-(PyBO)$_4$ (right) under UV light (λ_{em}= 365 nm) in air for different times. quartz plates (1 cm × 3 cm).

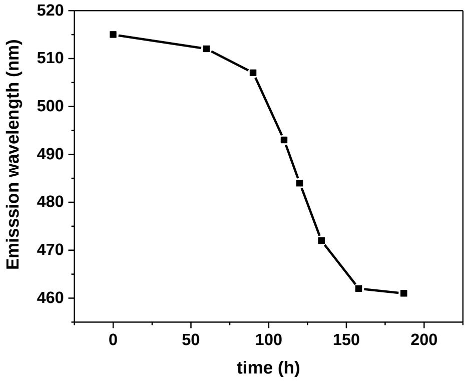

Supplementary Fig. 7. The maximum emission wavelength versus time. The plot of maximum emission wavelength of *R*-M-(PyBO)$_4$ versus time.

8. CPL spectra of *R/S*-M-(PyBO)$_4$ at different time.

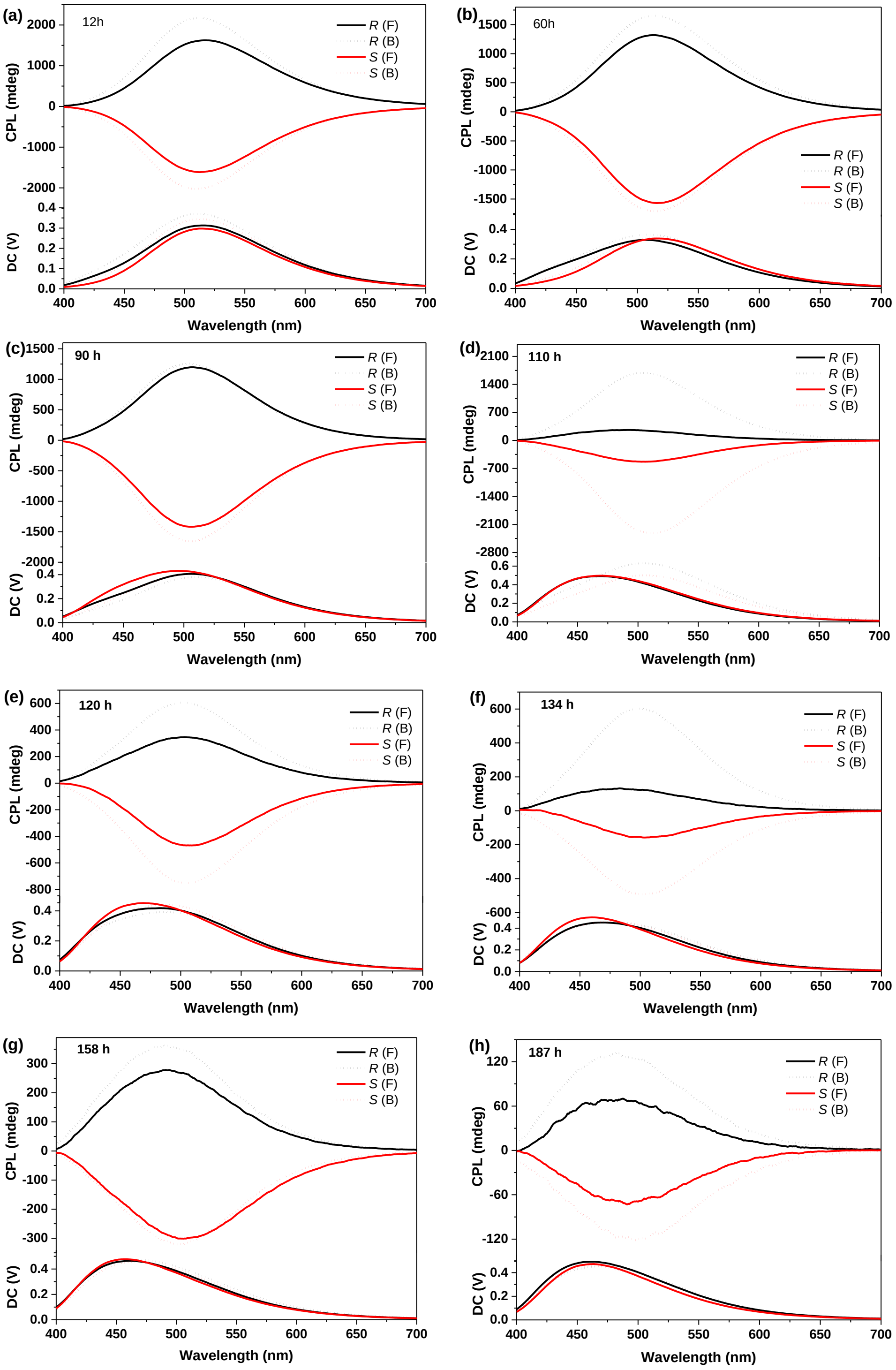

Supplementary Fig. 8. CPL spectra versus time. CPL spectra of chiral co-assembly *R/S*-M-(PyBO)$_4$ in the air for different time (**a**: 12 h; **b**: 60 h; **c**: 90 h; **d**: 110 h; **e**: 120 h; **f**: 134 h; **g**: 158 h; **h**: 187 h) (spin-coated from 40 mg/mL toluene solution, λ_{ex} = 330 nm, the films facing toward the detector: Frontward, F; away from the detector: Backward, B).

9. POM and Optical microscope images of *R/S*-M and PyBO films spin-coated from toluene solution.

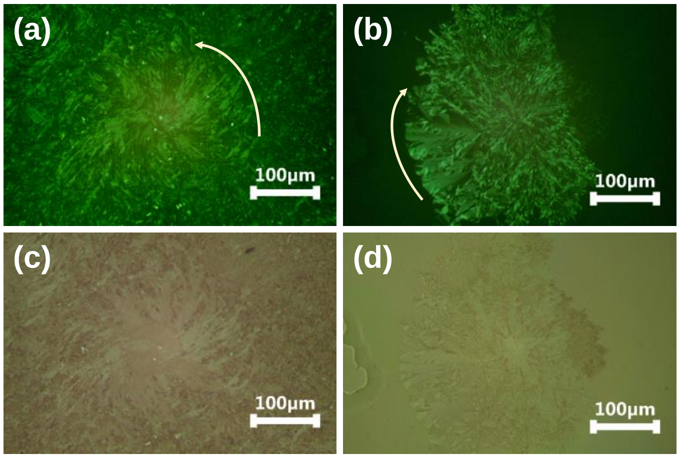

Supplementary Fig. 9. POM and optical microscope images of *R/S*-M. (a) POM images and **(c)** Optical microscope images of *R*-M; **(b)** POM images and **(d)** Optical microscope images of *S*-M. (spin-coated films after 20 min). All films were spin-coated from toluene solutions (40 mg/mL) on quartz plates (1 cm × 3 cm) (1000 N/s, 30 s).

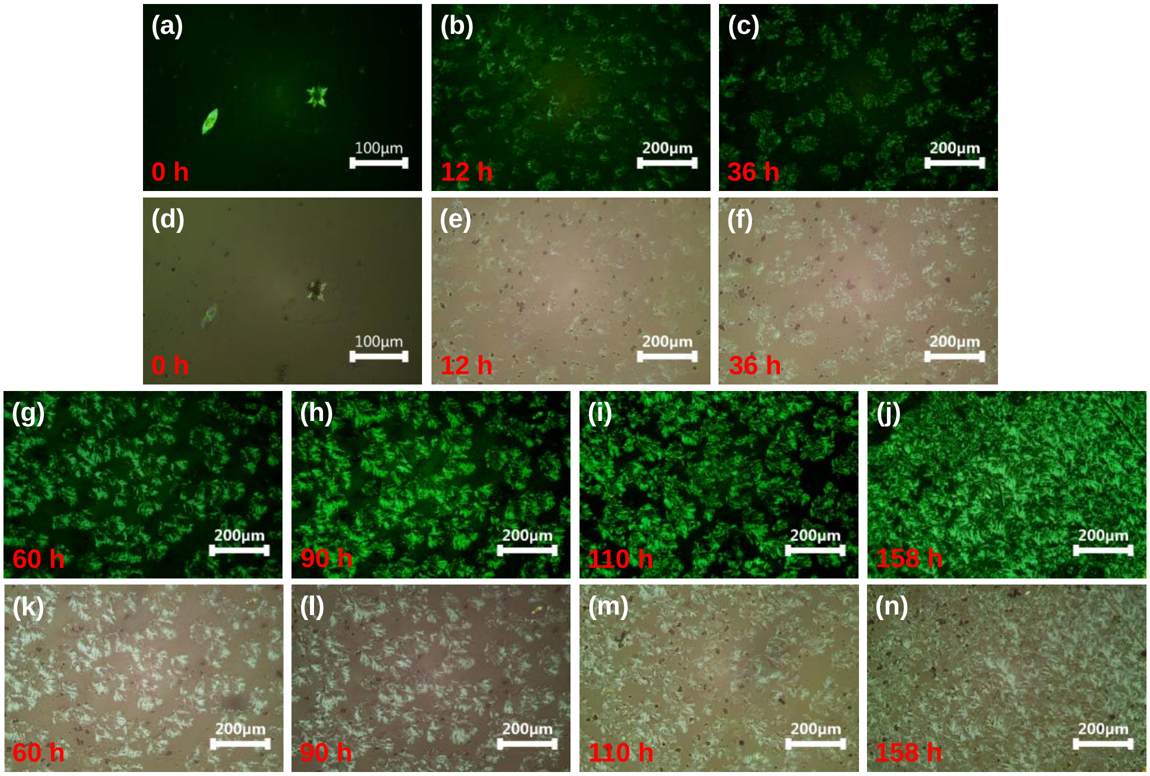

Supplementary Fig. 10. POM and optical microscope images of PyBO. (a-c, g-j) POM images and **(d-f, k-n)** Optical microscope images of PyBO. All films were spin-coated from toluene solutions (40 mg/mL) on quartz plates (1 cm × 3 cm) (1000 N/s, 30 s).

10. The plot of maximum emission wavelength of *R*-M-(PyBO)$_4$ versus spherulite diameters.

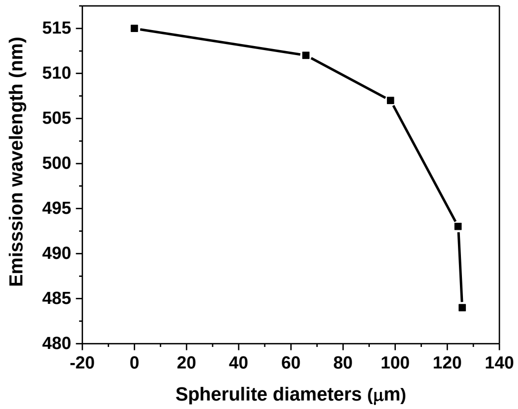

Supplementary Fig. 11. The maximum emission wavelength versus spherulite diameters. The plot of maximum emission wavelength of *R*-M-(PyBO)$_4$ versus spherulite diameters.

11. CPL spectra of *R*-M-(PyBO)$_4$-D/T.

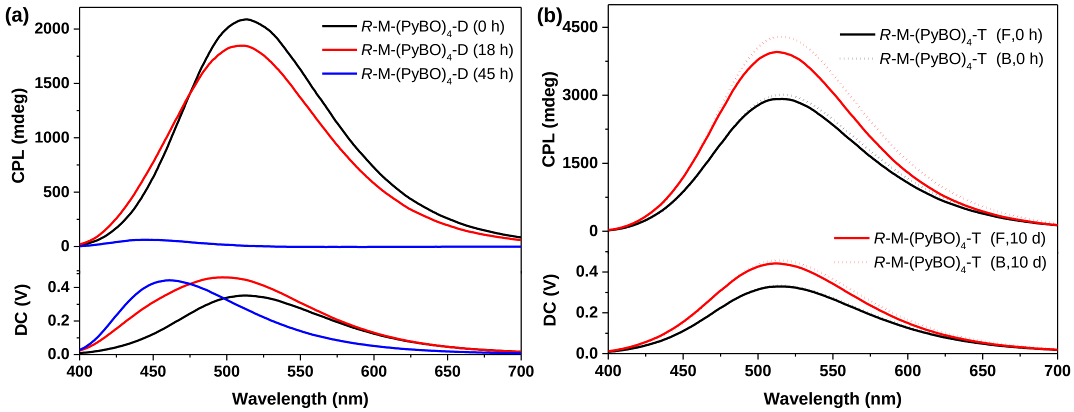

Supplementary Fig. 12. CPL spectra of *R*-M-(PyBO)$_4$-D/T. CPL spectra of **(a)** *R*-M-(PyBO)$_4$-D and **(b)** *R*-M-(PyBO)$_4$-T at different time. All spin-coated films spin-coated from 40 mg/mL solutions, the films facing toward the detector: Frontward, F; away from the detector: Backward, B (λ_{ex} = 330 nm).

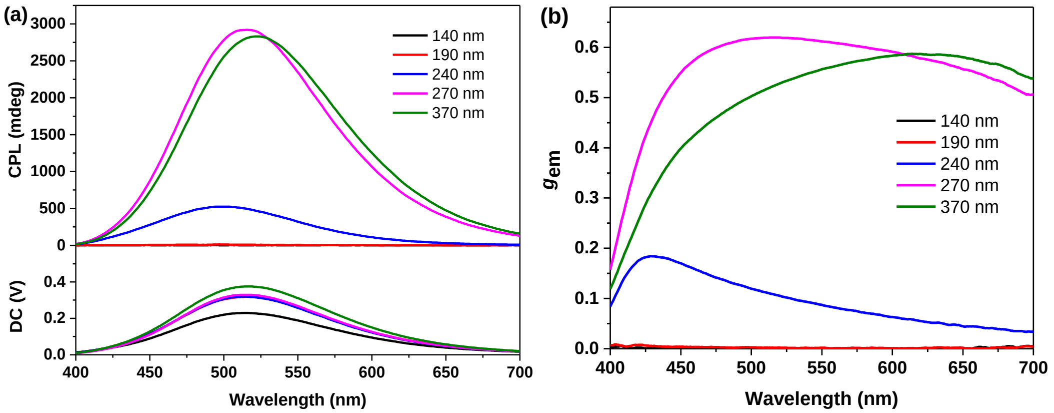

Supplementary Fig. 13. Chiral optical properties versus thickness. (a) CPL spectra and **(b)** the plot g_{em} values of *R*-M-(PyBO)$_4$-T spin-coated from THF solutions of different concentrations (10, 20, 30, 40, 50 mg/mL, respectively) (λ_{ex} = 330 nm).

12. POM and Optical microscope images of *R*-M-(PyBO)$_4$-D/T.

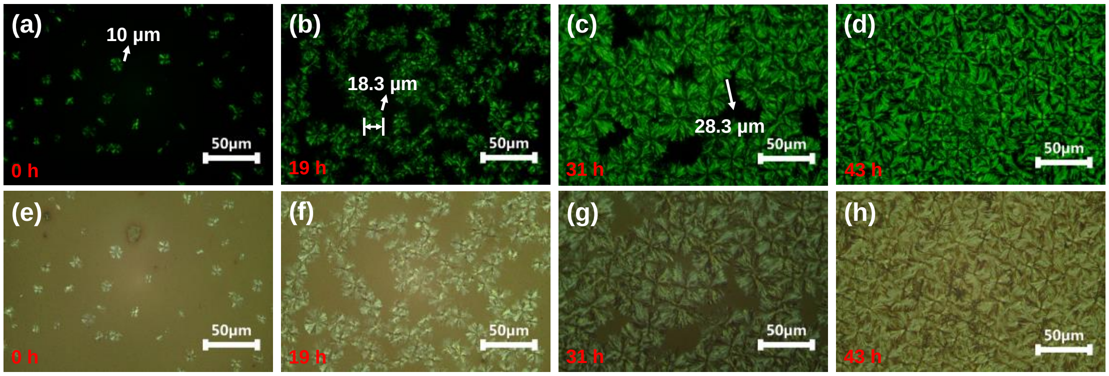

Supplementary Fig. 14. POM and optical microscope images of *R*-M-(PyBO)$_4$-D. (a-d) POM images and **(e-h)** Optical microscope images of *R*-M-(PyBO)$_4$-D films spin-coated from DCM solutions (40 mg/mL) at different time.

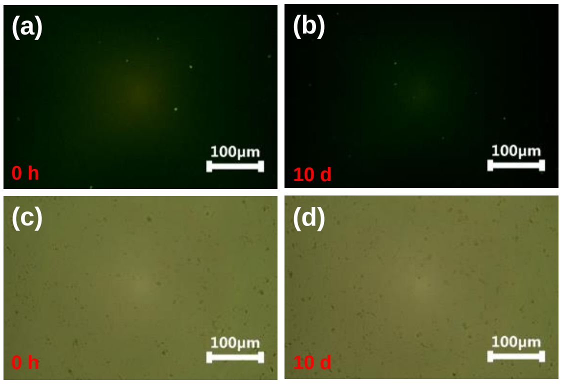

Supplementary Fig. 15. POM and optical microscope images of *R*-M-(PyBO)$_4$-T. (a,b) POM images and **(c,d)** Optical microscope images of *R*-M-(PyBO)$_4$-T films spin-coated from THF solutions (40 mg/mL) at different time.

13. SEM images.

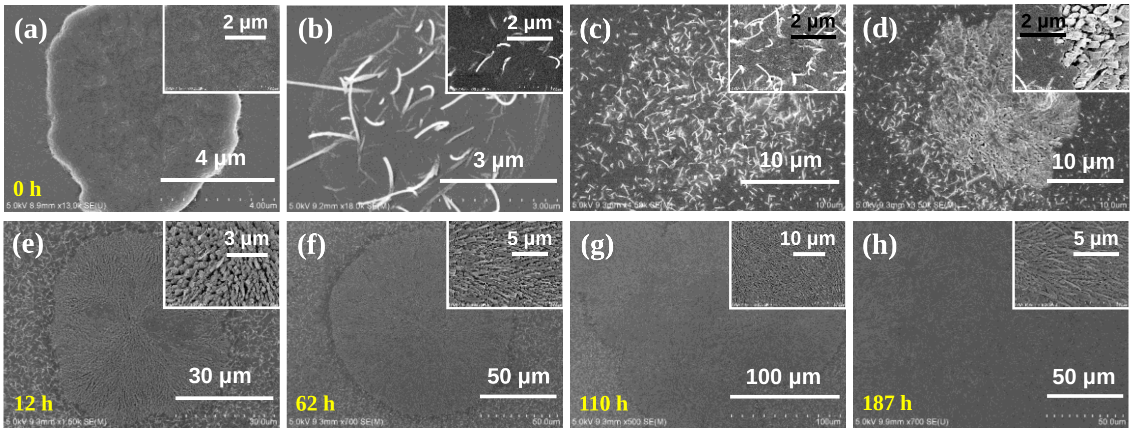

Supplementary Fig. 16. SEM images of *R*-M-(PyBO)$_4$ spin-coated films at different time. a was tested at 0 h; **b-e:** at 12 h; **f:** at 62 h; **g:** at 110 h; **h:** at 187 h. All spin-coated films from 40 mg/mL toluene solutions on quartz plates (1 cm × 3 cm) (1000 N/s, 30 s).

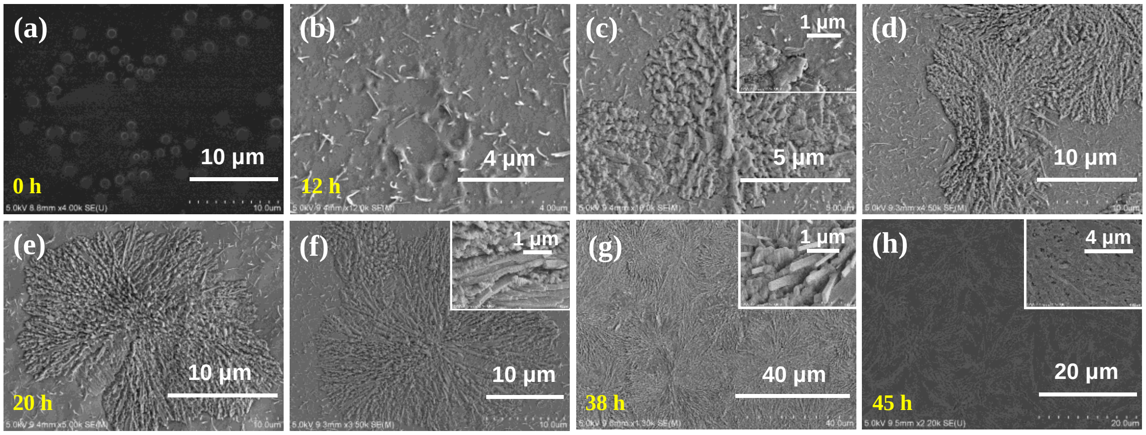

Supplementary Fig. 17. SEM images of *R*-M-(PyBO)$_4$-D at different time. a was tested at 0 h; **b-d**: at 12 h; **e-f**: at 20 h; **g:** at 38 h; **h:** at 45 h. All spin-coated films from 40 mg/mL DCM solutions on quartz plates (1 cm × 3 cm) (1000 N/s, 30 s).

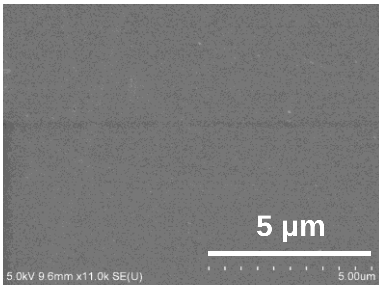

Supplementary Fig. 18. SEM image of *R*-M-(PyBO)$_4$-T. The SEM image were tested after 10 days later. The film spin-coated from 40 mg/mL THF solutions on quartz plates (1 cm × 3 cm) (1000 N/s, 30 s).

14. XRD patterns.

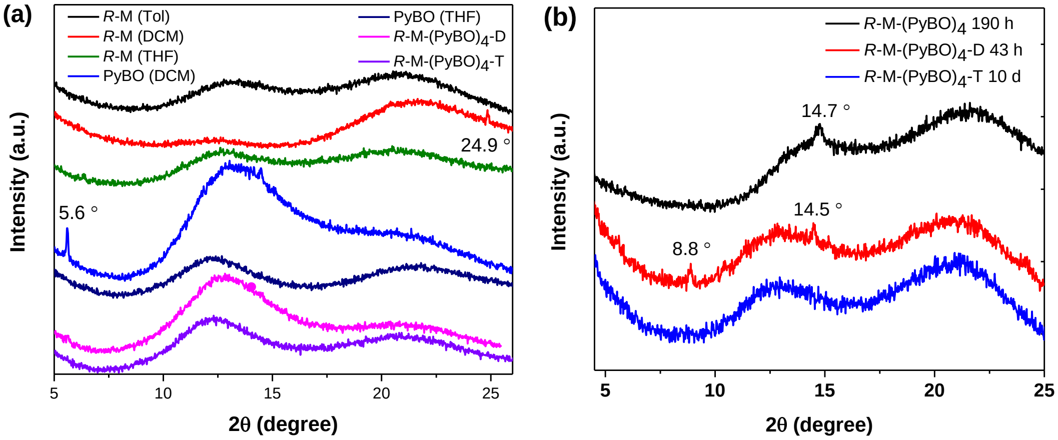

Supplementary Fig. 19. XRD patterns. (a) XRD patterns of *R*-M, PyBO and *R*-M-(PyBO)$_4$ fleshly films; **(b)** XRD patterns of *R*-M-(PyBO)$_4$, *R*-M-(PyBO)$_4$-D and *R*-M-(PyBO)$_4$-T in air for some time (h).

15. Comparison of the part of chiral excimer system.

Supplementary Table 2. Comparison of the part of chiral excimer system

Compound	state	λ_{em} (nm)	$\|g_{em}\|$	reference
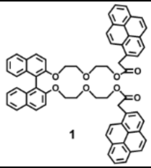	$CHCl_3$ solution	480	7.8×10^{-4}	*Chem. Commun.* **2014**, *50*, 13228
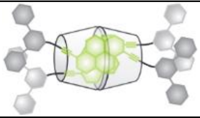	ammonia-containing H_2O at ca. pH 9.5.	528	1.5×10^{-2}	*Angew. Chem. Int. Ed.* **2014**, *53*, 14392
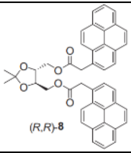	$CHCl_3$ solution	460	8.9×10^{-4}	*Chem. Commun.* **2015**, *51*, 8237
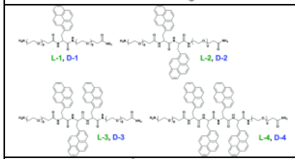	$CHCl_3$ solution	460	$0.3 \sim 1.0 \times 10^{-2}$	*Org. Biomol. Chem.* **2015**, *13*, 11426.
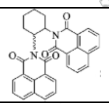	MeOH solution	470	3.7×10^{-2}	*Chem. Eur. J.* **2016**, *22*, 9519.
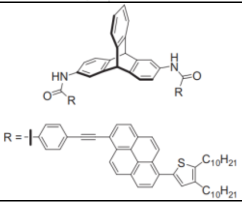	hexane/THF (95:5) solution	520	1.4×10^{-3}	*Org. Biomol. Chem.* **2017**, *15*, 8440.
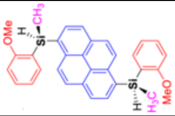	CH_2Cl_2 solution	500	8×10^{-3}	*J. Org. Chem.* **2017**, *82*, 6108.
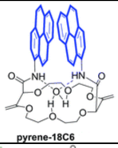	CH_3CN with $Ba(ClO_4)_2$.	490	9×10^{-3}	*Chem. Sci.* **2018**, *9*, 7043.
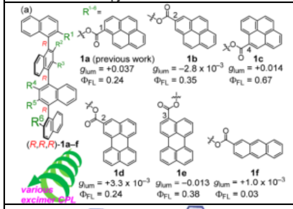	CH_2Cl_2 or THF solution	Blue to orange	$0.1 \sim 1.4 \times 10^{-2}$	*J. Am. Chem. Soc.* **2019**, *141*, 6185.
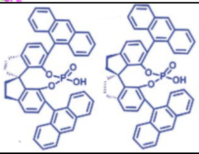	THF/H_2O (1/9) solution	460	2.9×10^{-2}	*Chem. Sci.* **2019**, *10*, 6821.

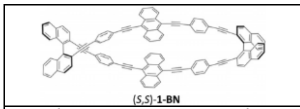	$CHCl_3$ solution	680	1.1×10^{-2}	*Chem. Eur. J.* **2019**, *25*, 9211.
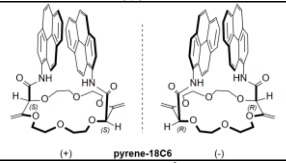	CH_3CN solution	490	9×10^{-3}	*Angew. Chem. Int. Ed.* **2019**, *58*, 6952.
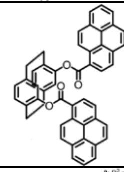	KBr-pellet	513	3.9×10^{-3}	*RSC Adv*. **2020**, *10*, 11335.
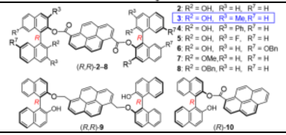	Toluene or DMSO solution	538	1.2×10^{-2}	*J. Am. Chem. Soc.* **2020**, *142*, 1774.
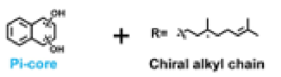	2 % Planar anthracene in Nap2 (molar ratios)	406	5.2×10^{-2}	*Angew. Chem. Int. Ed.* **2021**, *60*, 3745.
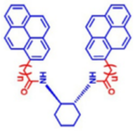	DMF solution	493~474 nm	$0.3\text{~}2.64 \times 10^{-2}$	*Angew. Chem. Int. Ed.* **2021**, *60*, 19451.
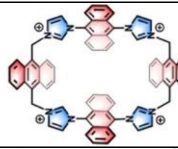	with ATP (0-1.5 equiv) in H_2O solution	550	1×10^{-2}	*Angew. Chem. Int. Ed.* **2021**, *60*, 15354.
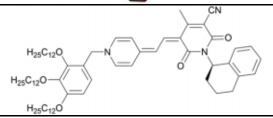	MCH solution	700	1.1×10^{-2}	*Chem. Sci.* **2021**, *12*, 12302.
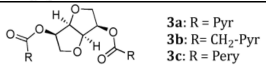	CH_2Cl_2 solution	500	9.6×10^{-3}	*Chem. Eur. J.* **2022**, e202104226. (10.1002/chem.202104226)
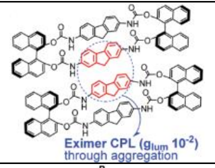	THF solution	370	1.1×10^{-2}	*Chem. Commun.* **2022**, *58*, 1029.
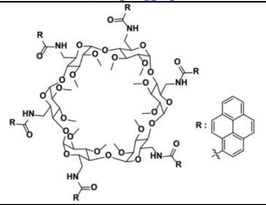	CH_2Cl_2 solution	486	1.2×10^{-2}	*Angew. Chem. Int. Ed.* **2022**, *61*, e202114700. (10.1002/ange.202114700)

16. ^{1}H and ^{13}C NMR Spectra of Compounds.

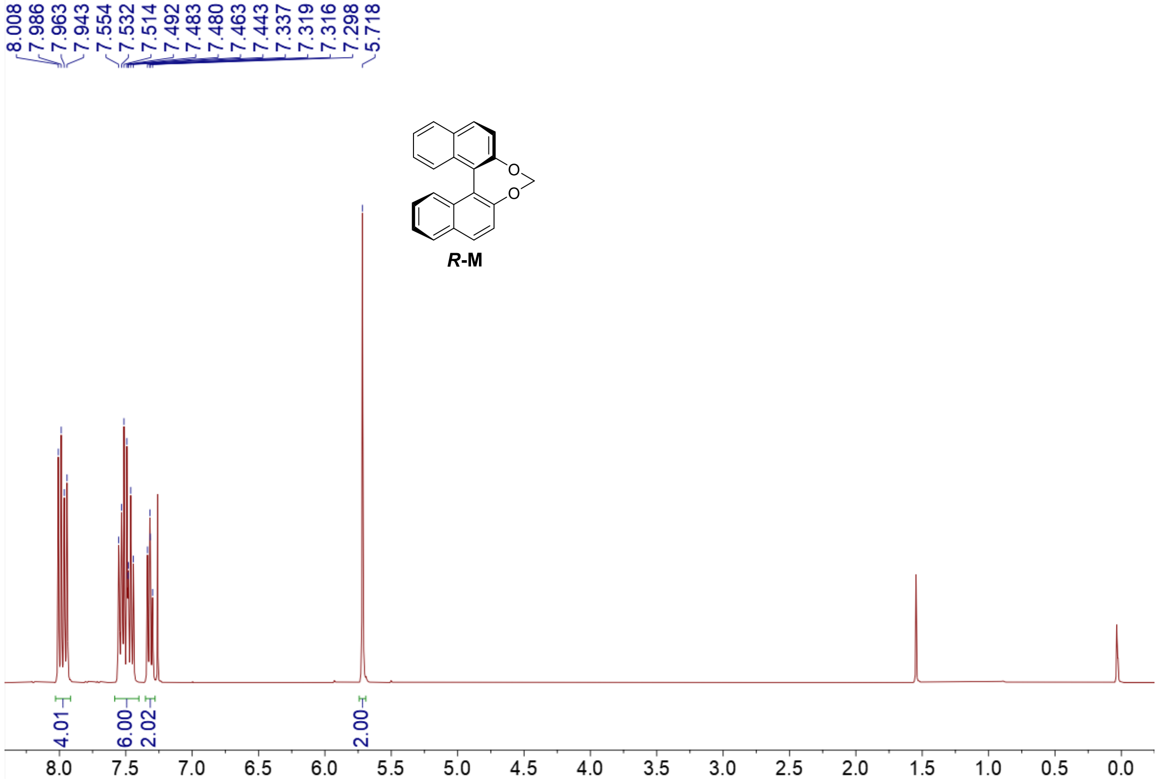

Supplementary Fig. 20. ^{1}H NMR spectrum. ^{1}H NMR spectrum of *R*-M (400 MHz, $CDCl_3$).

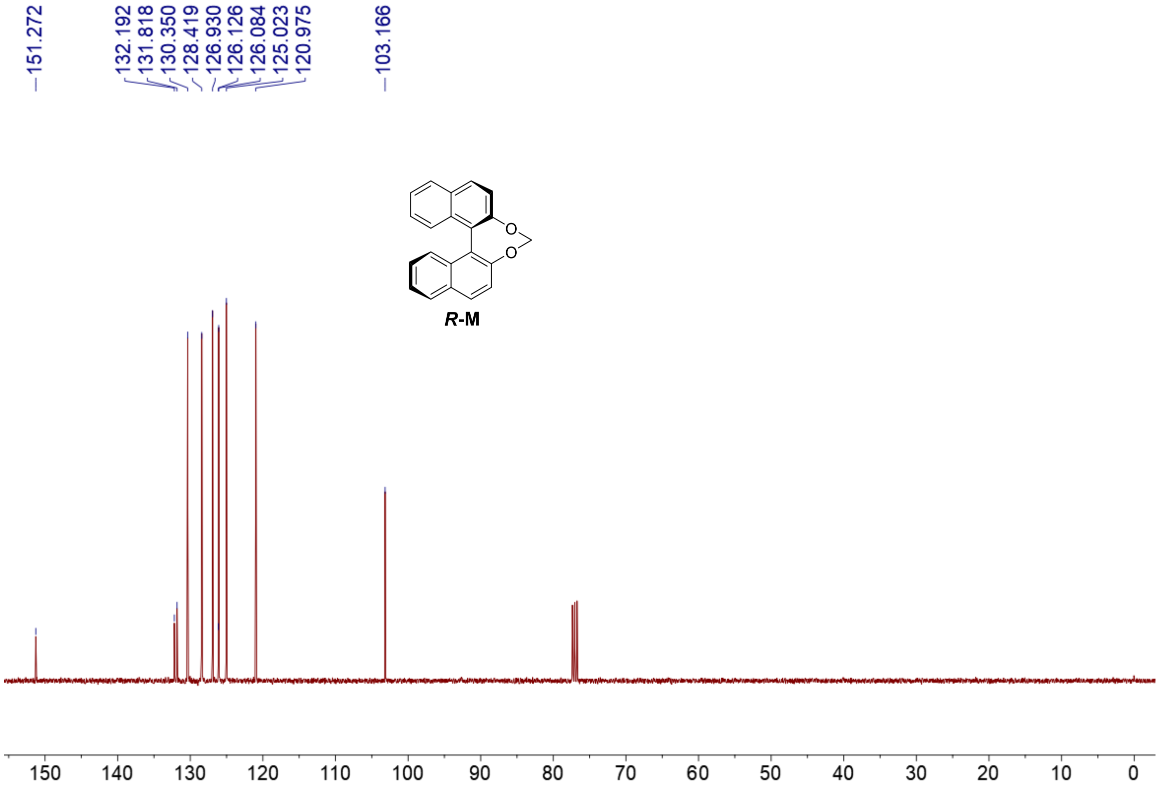

Supplementary Fig. 21. ^{13}C NMR spectrum. ^{13}C NMR spectrum of *R*-M (100 MHz, $CDCl_3$).

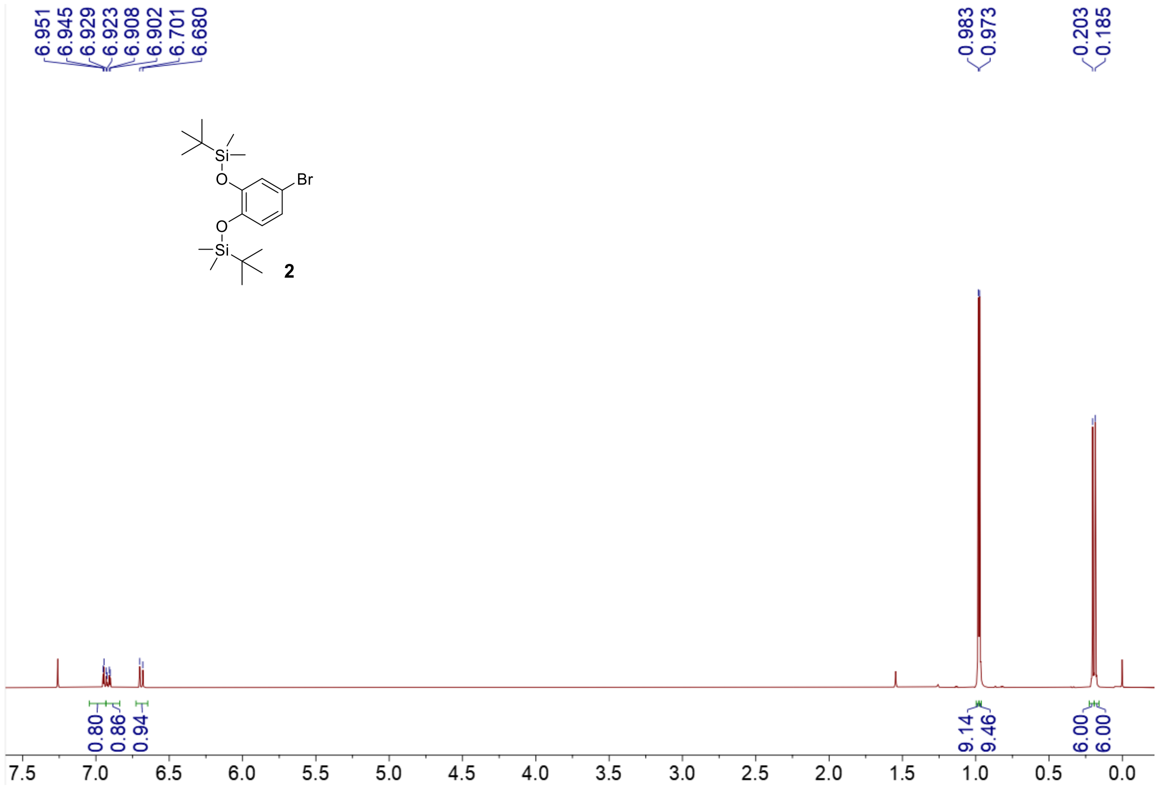

Supplementary Fig. 22. ^{1}H NMR spectrum. ^{1}H NMR spectrum of compound **2** (400 MHz, $CDCl_3$).

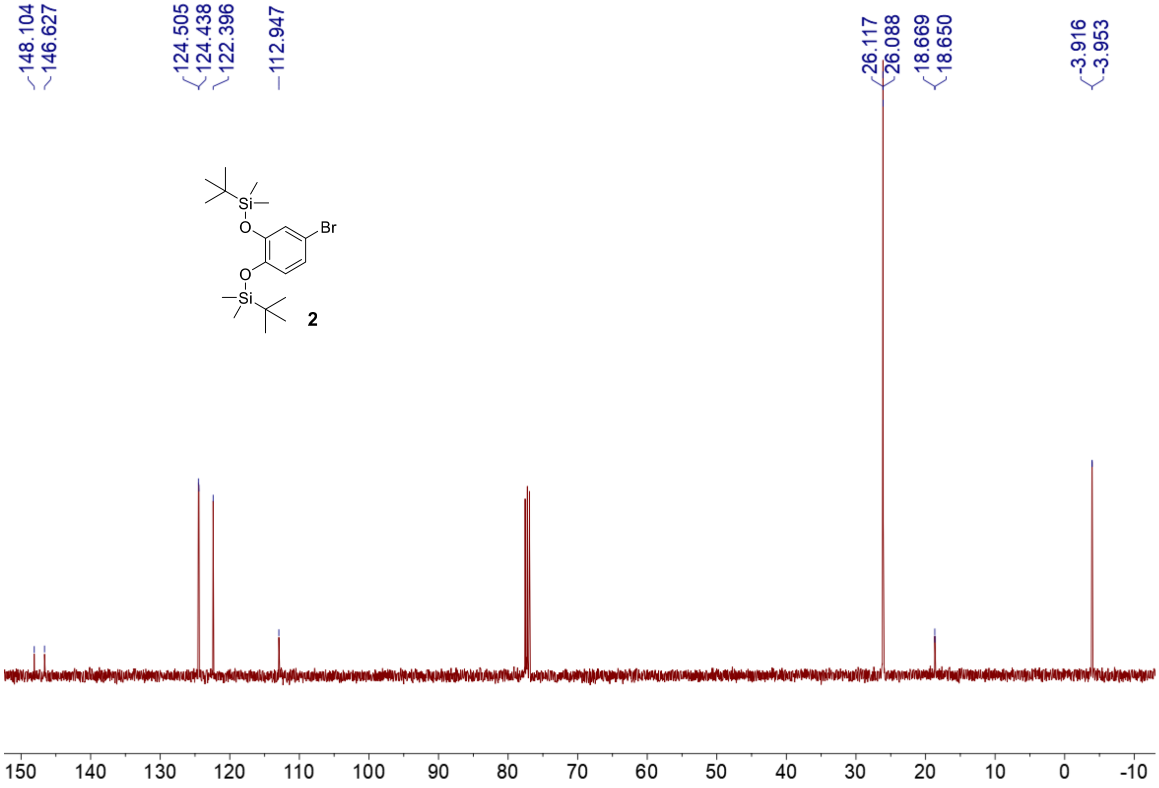

Supplementary Fig. 23. ^{13}C NMR spectrum. ^{13}C NMR spectrum of compound **2** (100 MHz, $CDCl_3$).

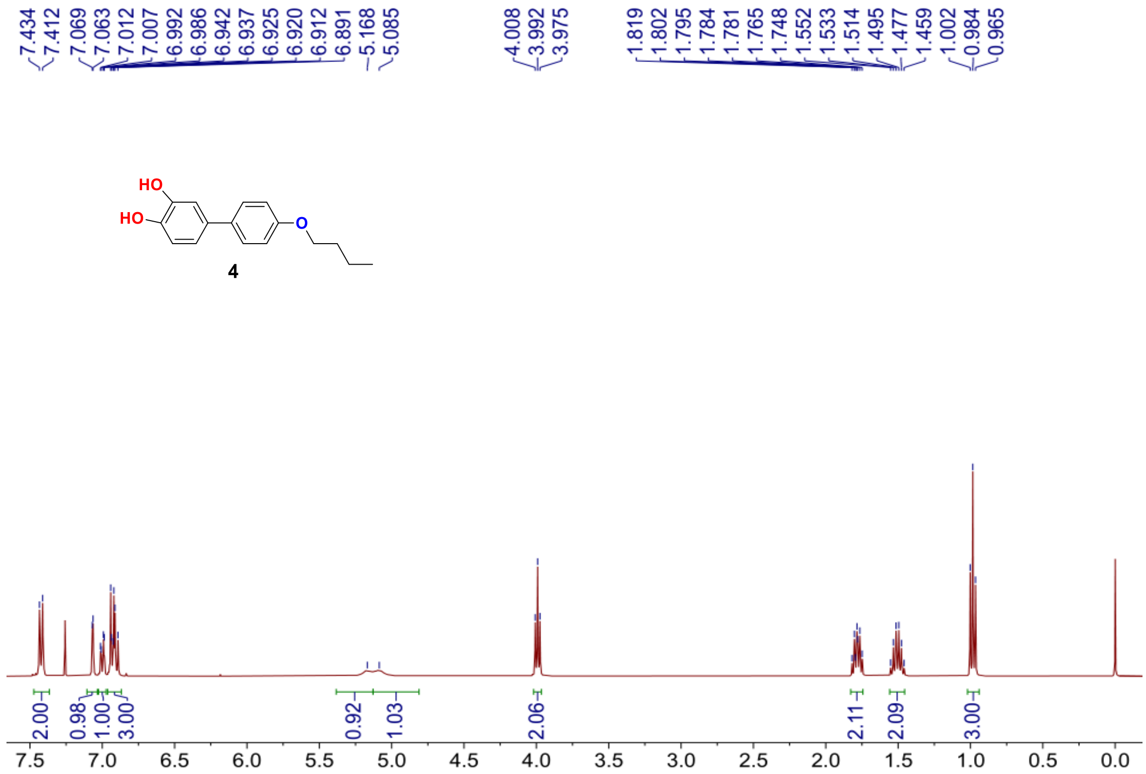

Supplementary Fig. 24. ^{1}H NMR spectrum. ^{1}H NMR spectrum of compound **4** (400 MHz, $CDCl_3$).

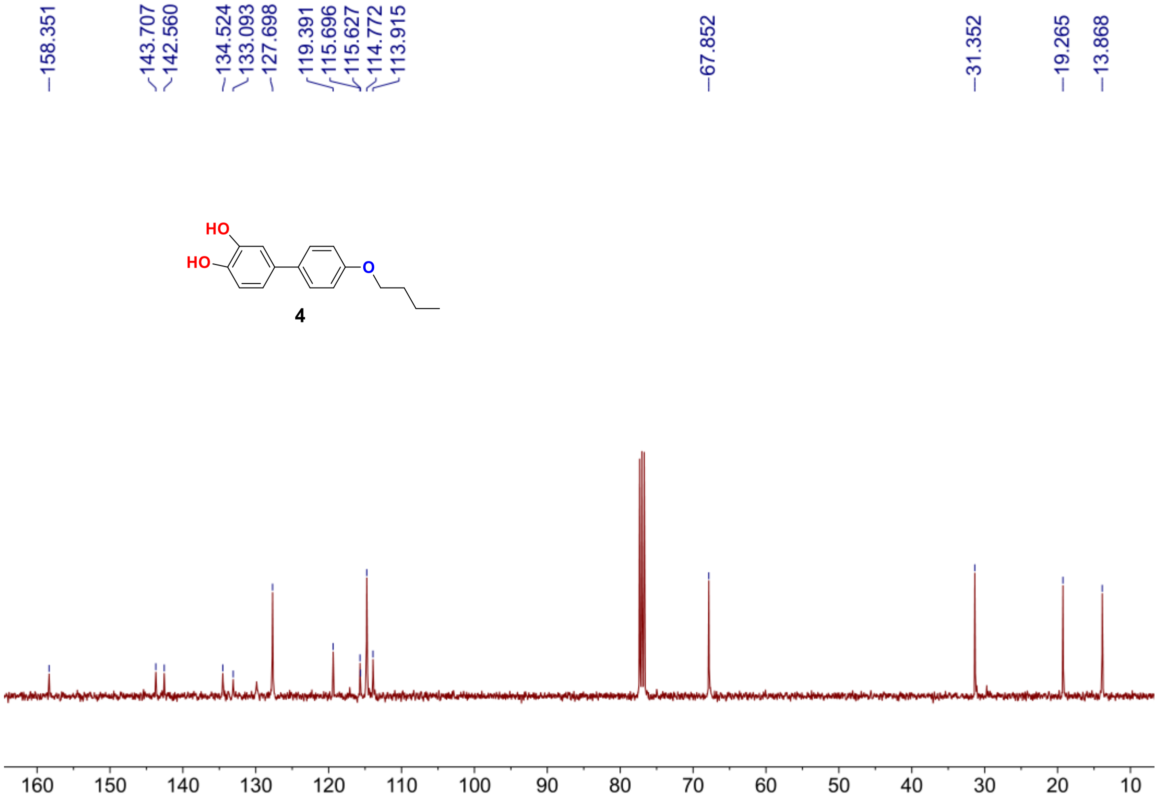

Supplementary Fig. 25. ^{13}C NMR spectrum. ^{13}C NMR spectrum of compound **4** (100 MHz, $CDCl_3$).

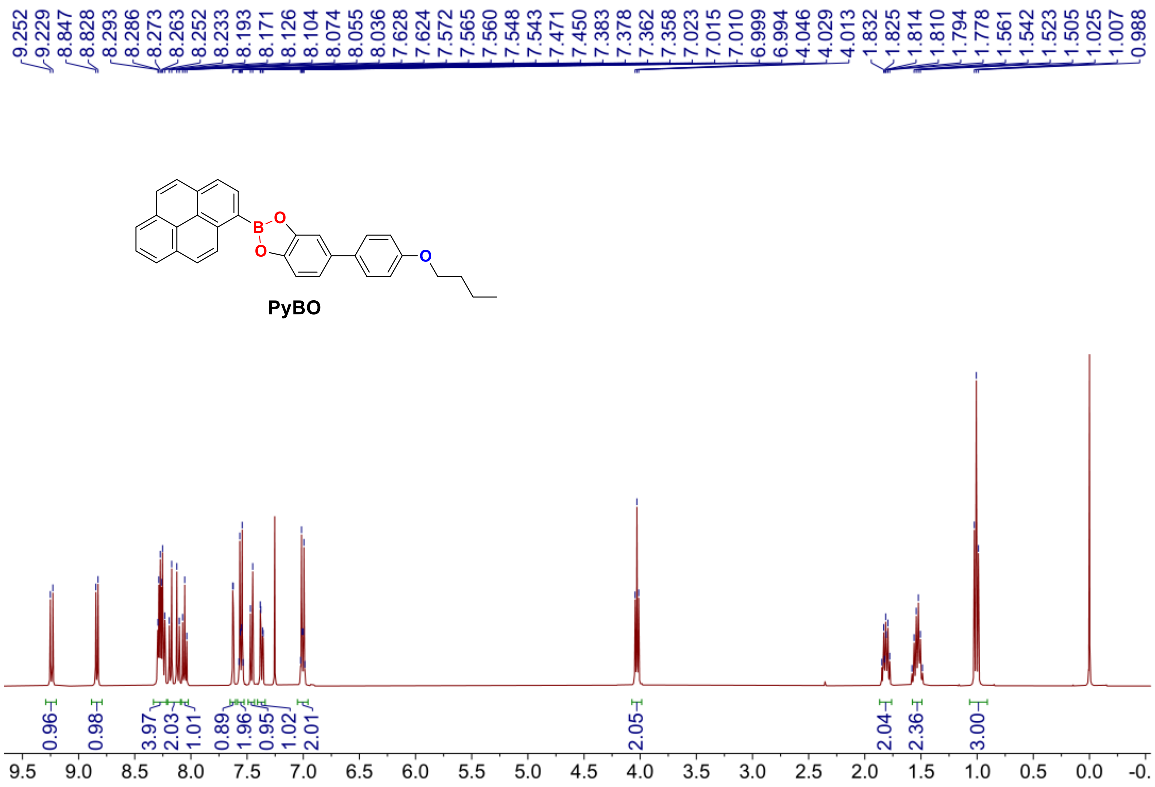

Supplementary Fig. 26. ^{1}H NMR spectrum. ^{1}H NMR spectrum of PyBO (400 MHz, $CDCl_3$).

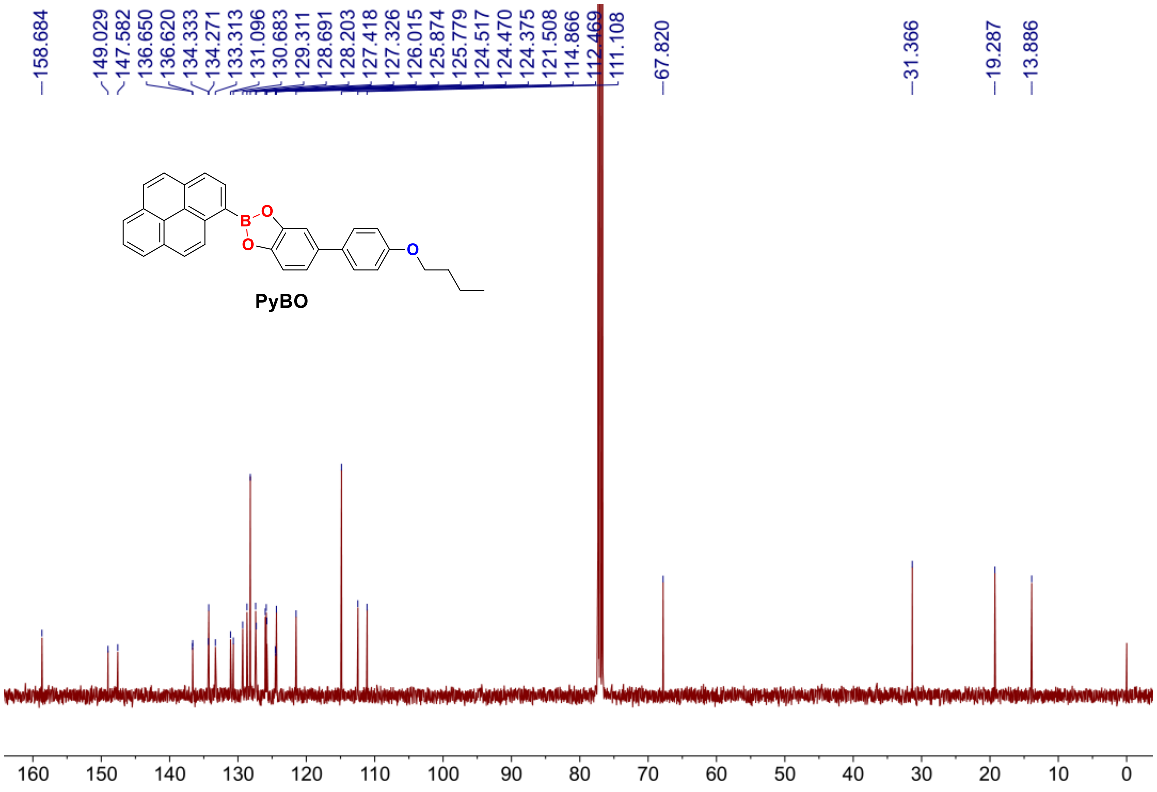

Supplementary Fig. 27. ^{13}C NMR spectrum. ^{13}C NMR spectrum of PyBO (100 MHz, $CDCl_3$).

17. Reference

1. Li, Y., Urbas, A. ＆ Li, Q. Reversible light-directed red, green, and blue reflection with thermal stability enabled by a self-organized helical superstructure. *J. Am. Chem. Soc.* **134,** 9573-9576 (2012).

2. Zhang, Y. et al. Inverted circularly polarized luminescence behavior induced by helical nanofibers through chiral co-assembly from achiral liquid crystal polymers and chiral inducers. *ACS Nano* **16,** 3173-3181 (2022).

3. Schrçder, P. et al. Neuritogenic militarinone-inspired 4-hydroxypyridones target the stress pathway kinase MAP4K4. *Angew. Chem. Int. Ed.* **54,** 12398-12403 (2015).
